# Supplementary figures and images for: Deprivation of dietary fiber enhances susceptibility of mice to cryptosporidiosis
Source: PLoS Negl Trop Dis. 2019 Sep 27;13(9):e0007411. doi: 10.1371/journal.pntd.0007411 (PMC6785118; doi:10.1371/journal.pntd.0007411)

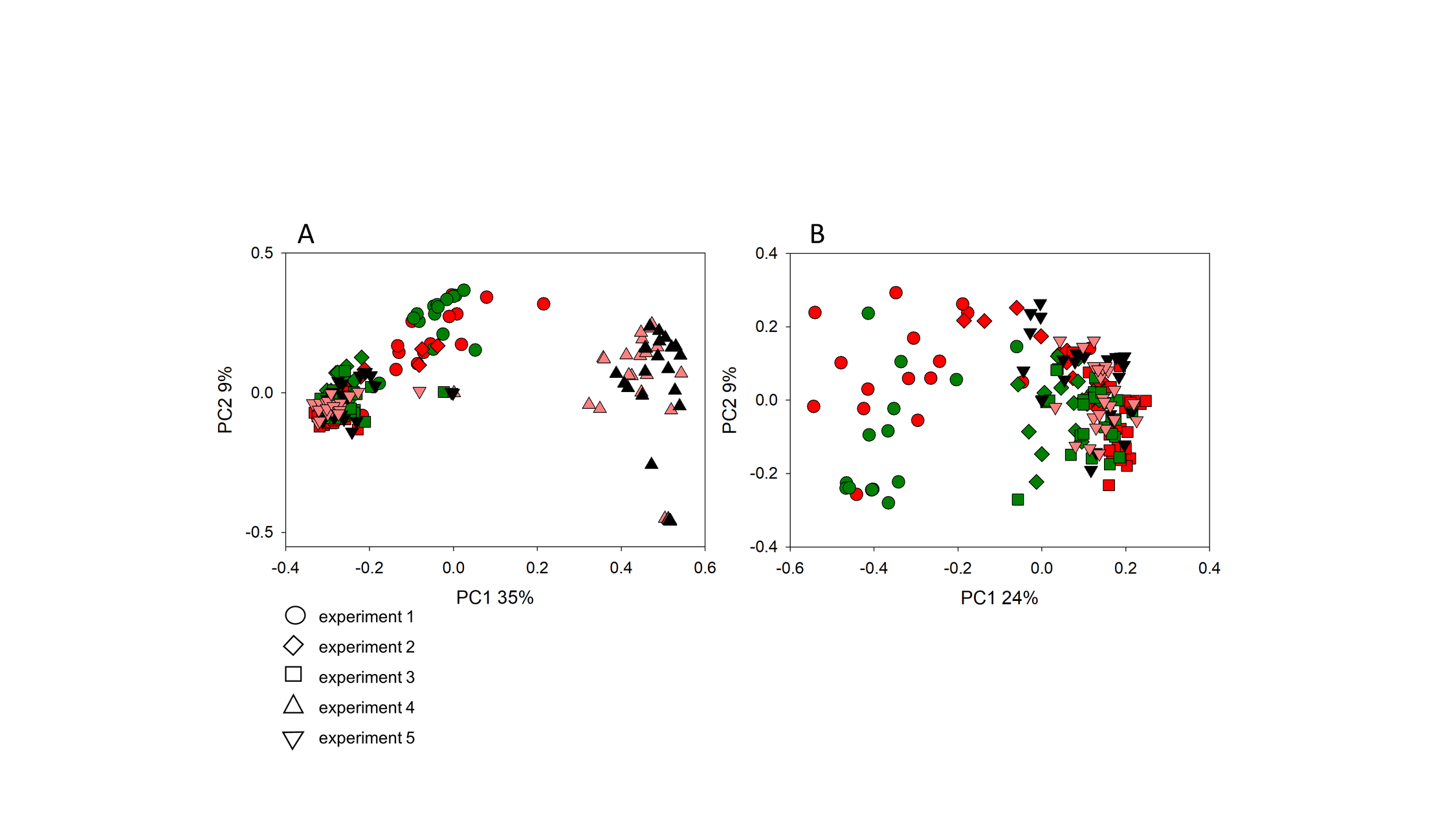

Supplement: S1 Fig — Each data point represents a fecal sample from one mouse. Samples collected over the entire duration of the experiments are included. A. All experiments (n = 212); B. Experiment 4 (antibiotic pre-treatment) excluded (n = 162) to de-compress the plot and visualize clustering of samples from the remaining experiments. Symbols indicate experiment as shown in the key. Color indicates treatment as follows: red, no-fiber diet; green, medium-fiber diet; pink, antibiotics followed by prebiotics; black, antibiotics only. (TIF) [file pntd.0007411.s001.tif]

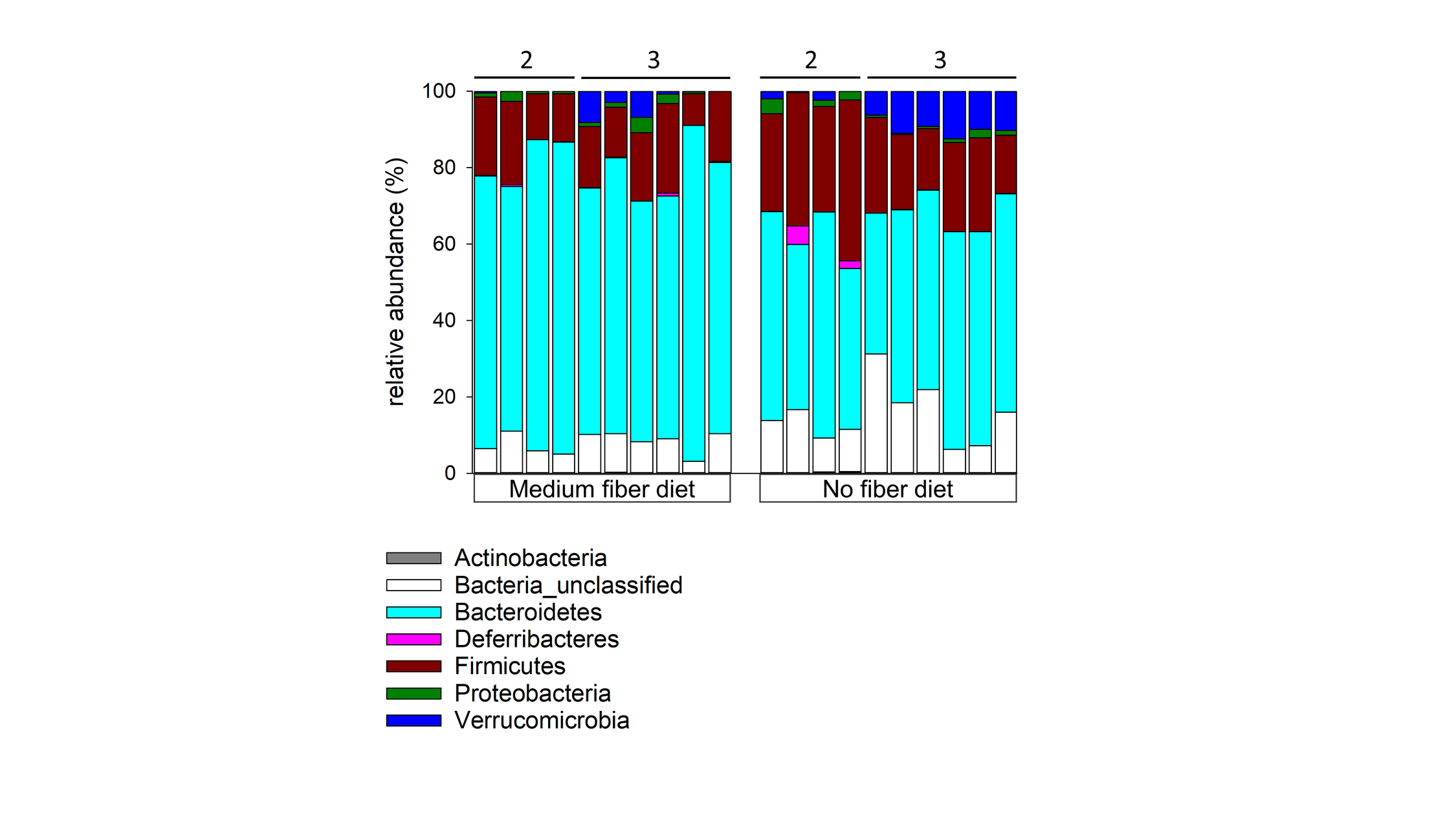

Supplement: S2 Fig — Each bar represents one sample collected from one mouse. The experiment number is indicated uppermost. (TIF) [file pntd.0007411.s002.tif]
